# Pifithrin-µ Induces Stress Granule Formation, Regulates Cell Survival, and Rewires Cellular Signaling

**DOI:** 10.3390/cells13110885

**Published:** 2024-05-21

**Authors:** Hicham Mahboubi, Henry Yu, Michael Malca, David McCusty, Ursula Stochaj

**Affiliations:** 1 Department of Physiology, McGill University, Montreal, QC H3G 1Y6, Canadahenry.yu2@mail.mcgill.ca (H.Y.); michael.malca@mail.mcgill.ca (M.M.);; 2 Quantitative Life Sciences Program, McGill University, Montreal, QC H3G 1Y6, Canada

**Keywords:** pifithrin-µ, stress granules, eIF2α phosphorylation, 5′-AMP-activated protein kinase (AMPK), protein kinase Akt

## Abstract

(1) Background: Stress granules (SGs) are cytoplasmic protein-RNA condensates that assemble in response to various insults. SG production is driven by signaling pathways that are relevant to human disease. Compounds that modulate SG characteristics are therefore of clinical interest. Pifithrin-µ is a candidate anti-tumor agent that inhibits members of the hsp70 chaperone family. While hsp70s are required for granulostasis, the impact of pifithrin-µ on SG formation is unknown. (2) Methods: Using HeLa cells as model system, cell-based assays evaluated the effects of pifithrin-µ on cell viability. Quantitative Western blotting assessed cell signaling events and SG proteins. Confocal microscopy combined with quantitative image analyses examined multiple SG parameters. (3) Results: Pifithrin-µ induced *bona fide* SGs in the absence of exogenous stress. These SGs were dynamic; their properties were determined by the duration of pifithrin-µ treatment. The phosphorylation of eIF2α was mandatory to generate SGs upon pifithrin-µ exposure. Moreover, the formation of pifithrin-µ SGs was accompanied by profound changes in cell signaling. Pifithrin-µ reduced the activation of 5′-AMP-activated protein kinase, whereas the pro-survival protein kinase Akt was activated. Long-term pifithrin-µ treatment caused a marked loss of cell viability. (4) Conclusions: Our study identified stress-related changes in cellular homeostasis that are elicited by pifithrin-µ. These insights are important knowledge for the appropriate therapeutic use of pifithrin-µ and related compounds.

## 1. Introduction

Stress granules (SGs) are transient cytoplasmic RNA-protein condensates that form in response to various insults. During stress, SG formation can promote adaptation and cell survival [1,2,3]. While the cell type and stressor determine the molecular composition of SGs [2], several granule components are commonly present. These include translationally stalled mRNAs, RNA-binding proteins, and key regulators of cell fate [4,5].

The assembly of canonical SGs requires the phosphorylation of translation initiation factor eIF2α on Ser51 (S51). This modification destabilizes polysomes and liberates mRNAs, which become available for binding to SG nucleators, such as G3BP1 [6,7]. Several signaling routes control SG formation and thereby affect cell survival. Specific examples are the energy sensors 5′-AMP-activated protein kinase (AMPK) and mTORC1, PI3 kinase, O-GlcNAc transferase, and the RhoA/Rock1 pathway [5,8,9,10,11,12].

Additional cellular regulators add further complexity to the stress response through the control of SG assembly and dissolution. Molecular chaperones in particular are critical for granule homeostasis [13,14,15,16,17], referred to as granulostasis. Specifically, hsp70s prevent the aggregation of SG-nucleating proteins [18], facilitate SG disassembly [19], and maintain overall granulostasis [17,20].

Given their importance for cellular homeostasis and stress survival, hsp70s have emerged as targets for therapeutic intervention. Consequently, pharmacological modulators of molecular chaperones continue to be assessed in clinical trials [21]. One lead compound for drug development is the small molecule pifithrin-µ (PFT-µ, also known as 2-phenylethynesulfonamide or PES [22]), which has been included in several patent applications [23,24,25]. PFT-µ inhibits the chaperone cycle for members of the hsp70 family (hsp70s and hsc70, here collectively called hsp70s). The compound interacts with the hsp70 substrate-binding domain in the carboxyl-terminal portion of the chaperone [26,27] and covalently modifies cysteine residues of hsp70 [28]. To date, PFT-µ represents an established inhibitor of hsp70s that has been widely used in pre-clinical studies. At the cellular level, PFT-µ affects diverse cellular compartments and activities, including mitochondria, lysosomes, autophagy, and necrosis [29,30,31,32,33,34,35].

PFT-µ may deregulate the cellular redox balance, thus generating a stressful environment [34,36]. In addition, the compound compromises the association of p53 with mitochondria, which modulates apoptosis [37]. It should be noted that PFT-α, which has been extensively used to ablate p53 function, differs from PFT-µ in its effects on cellular homeostasis [37]. The current study focuses on PFT-µ.

Due to the various contributions of hsp70s to cell physiology, PFT-µ has been evaluated for therapeutic applications of health conditions which range from peripheral neuropathy [38,39] to cancer [30,40,41,42,43,44,45,46]. As a potential anti-cancer agent, PFT-µ enhances the anti-tumor effects of heat stress [47] and is effective in combination with hsp90 inhibitors [48,49].

Diverse signaling pathways regulate tumor cell survival and proliferation. The roles of AMPK [50,51,52] and the PI3 kinase/Akt pathway are especially well characterized [53,54,55]. Notably, there is crosstalk and negative feedback between AMPK and Akt signaling routes, especially under conditions of oxidative stress [56,57,58,59]. Heat shock proteins, particularly hsp70s, control this crosstalk [60].

Although important for the design of therapeutic regimens, the cellular effects of PFT-µ are not fully defined. To fill these knowledge gaps, we focused on the stress responses triggered by PFT-µ. Our study demonstrates that PFT-µ induces the formation of *bona fide* SGs. This process relies on eIF2α phosphorylation. In addition, PFT-µ significantly alters the signaling through AMPK and Akt kinases, and long-term PFT-µ treatment reduces cell viability. Taken together, we identified novel roles of PFT-µ in the regulation of three major pathways that are essential for the survival of deleterious conditions: SG formation, AMPK activation, and Akt signaling. Our study advances the understanding of the mechanism-of-action of the lead compound PFT-µ. This has important implications for the development of therapeutic applications.

## 2. Materials and Methods

### 2.1. Primary and Secondary Antibodies

All horseradish peroxidase (HRP)-coupled, fluorescently labeled, and secondary antibodies were generated in donkeys. They were affinity-purified and cross-absorbed against antibodies from multiple species (see Table 1 for details).

### 2.2. Cell Culture and Drug Treatments

HeLa cells were originally obtained from ATCC (American Type Culture Collection). Their characteristics are available through the Cellosaurus database (accession number: CVCL_0030) [61]. HeLa cells were cultured in Dulbecco’s modified Eagle’s medium (DMEM) with 8% fetal bovine serum (FBS) under standard tissue culture conditions (37 °C, 5% CO_2_).

Wildtype and eIF2αS51A mutant mouse embryonic fibroblasts (MEFs) were kindly provided by Dr. Koromilas, McGill University. The generation of knock-in MEFs has been published [62]. These MEFs carry a serine 51 to alanine mutation (S51A) in both eIF2α alleles. The cells encode human eIF2αS51A; an internal ribosome entry site (IRES) regulates the production of green fluorescence protein (GFP). The presence of GFP marks cells that synthesize the mutant eIF2αS51A protein. MEFs were cultured in DMEM supplemented with FBS and 2.5 μg/mL puromycin (Sigma, Oakville, ON, Canada), as described previously [5,62].

HeLa cells and MEFs were incubated with the vehicle DMSO or 50 µM PFT-µ for the times indicated in the figure legends. The final concentration of DMSO was 0.1%. Treatment with staurosporine (24 h, 1 µM final concentration) was used to induce apoptosis [63].

### 2.3. Preparation of Crude Cell Extracts

The protocol to generate crude cell extracts has been described in detail [64]. In brief, control or treated HeLa cells were scraped into 0.5× concentrated gel sample buffer and boiled at 95 °C. Proteins were then precipitated with trichloroacetic acid and resuspended in gel sample buffer (80 mM Tris-HCl, pH 8.0, 0.1 M dithiothreitol (DTT), 2% sodium dodecyl sulfate (SDS), 11.5% (*v*/*v*) glycerol, 0.002% bromophenol blue, supplemented with a cocktail of protease and phosphatase inhibitors) [65].

### 2.4. Western Blotting

Crude cell extracts were separated on 7.5–11% or 10–12% poly-acrylamide gels; Western blotting followed standard procedures [64]. All steps were carried out with gentle agitation. Blocking and antibody incubation for phospho-epitopes were performed with 1% bovine serum albumin (BSA)/50 mM NaF in Tris-buffered saline (TBS), containing 0.05% Tween20. For all other antibodies, blocking and incubation steps were conducted with 5% skim milk powder in TBS/0.05% Tween 20. After 1 h blocking at room temperature, filters were incubated with primary and secondary antibodies overnight at 4 °C. Dilutions of primary and secondary antibodies are listed in Table 1. Bound secondary antibodies were detected with enhanced chemiluminescence (ECL). ECL reagents were purchased from Amersham. Raw data for Western blots are included in the Supplementary File.

### 2.5. Immunolocalization

Immunofluorescent staining followed our published procedures [5,64,66]. In brief, cells grown on poly-lysine coated coverslips were treated, fixed, permeabilized, and blocked in 5% fetal bovine serum/phosphate buffered saline (PBS)/0.05% Tween20 (blocking solution). All subsequent steps were carried out in blocking solution. Primary antibodies were added overnight at the dilutions listed in Table 1. The following day, samples were washed and incubated with fluorescently labeled secondary antibodies (Table 1). After washing, DNA was stained with 1 µg/mL 4′,6-diamidino-2-phenylindole (DAPI). Coverslips were mounted and samples were inspected by fluorescence microscopy.

### 2.6. In Situ Hybridization

The detection of polyA^+^-containing messenger RNA (mRNA) was performed with oligo-dT50 labeled with 6-FAM (6-carboxyfluorescein; purchased from Bio Basic Inc., Markham, ON, Canada). We followed our detailed protocol, essentially as published [67,68]. Instead of transfer RNA, the current study used salmon sperm DNA at a final concentration of 100 µg/mL.

### 2.7. Confocal Microscopy and Quantitative Image Analysis

All protocols for image acquisition and quantification have been described by us in detail [9,64,68]. MetaXpress^®^ software (version 5 5.00.20, Molecular Devices, San Jose, CA, USA) was used to quantify SG parameters with our published procedures [68]. G3BP1 served as marker protein for SGs.

### 2.8. Cell Viability Assay

A resazurin (Acros Organics, ThermoFisher Scientific, Saint-Laurent, QC, Canada) reduction assay evaluated cell viability [69]. To this end, cells were grown in 96-well plates (Corning, MilliporeSigma, Oakville, ON, Canada), and cell viability was determined with CellTiter-Blue^®^ (Promega, Madison, WI, USA) according to the manufacturer’s recommendations. Fluorescence (560 ex/590 em) was measured 2 h after addition of the reagent using a Tecan Infinite M-1000 plate reader (Tecan, Männedorf, Switzerland). Background fluorescence of the medium was subtracted for each treatment condition.

### 2.9. Statistics

Statistical evaluation was carried out with a one-way analysis of variance (ANOVA) test combined with Bonferroni post hoc analysis. Differences were considered significant for *p* < 0.05. Details of the comparisons are provided in the figure legends.

## 3. Results and Discussion

### 3.1. Pifithrin-µ Induces Stress Granule Formation in the Absence of Other Stressors

Molecular chaperones, including members of the hsp70 family, contribute to the regulation of SG dynamics [13,18,19]. The hsp70 inhibitor PFT-µ has potent anti-cancer activities in cellular and mouse models (for example [70]). At the same time, several chemotherapeutic agents promote the assembly of SGs and thereby affect cancer cell survival [1]. These earlier observations provided the rationale to determine whether PFT-µ impinges on SG formation. HeLa cells were selected for our study because they have been used extensively to examine SG properties (for example [5,12,71,72]). The appropriate range of PFT-µ concentrations for HeLa cells has been determined previously [29,73,74]. These earlier results provided guidance for the experimental design described below.

**Figure 1:**
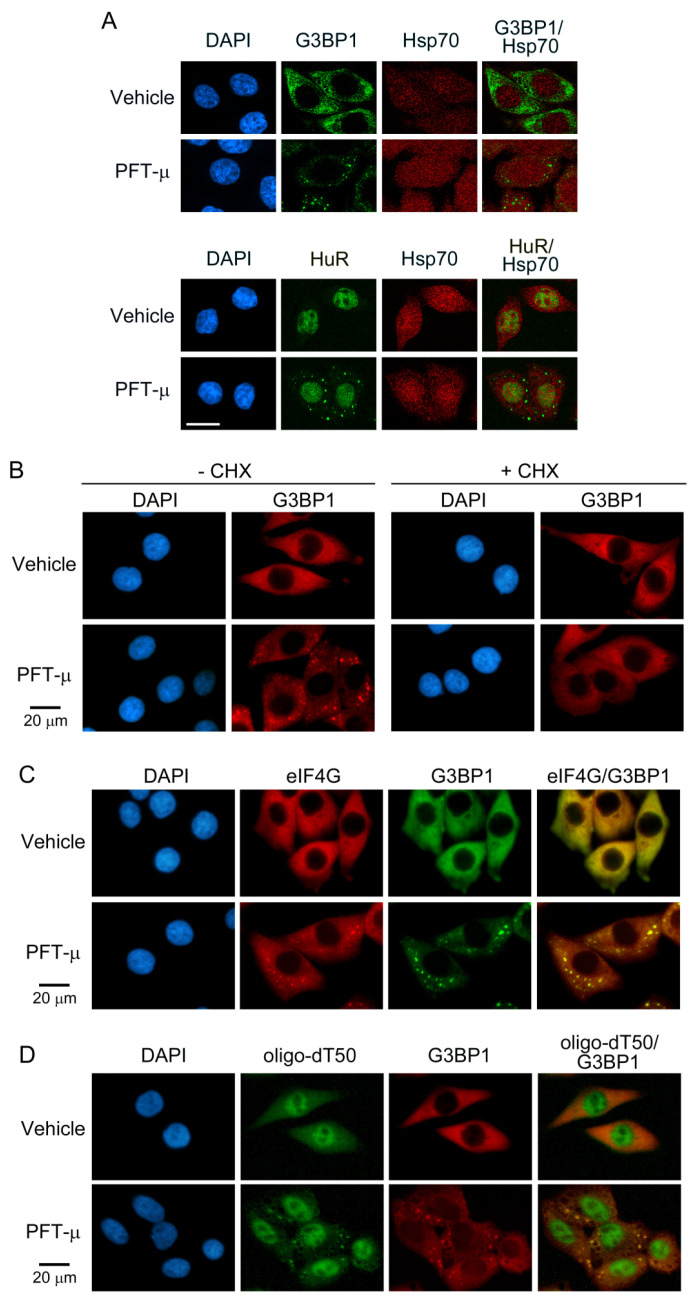
PFT-µ triggers the assembly of *bona fide* cytoplasmic stress granules. (**A**) HeLa cells were incubated with vehicle or PFT-µ (50 µM, 1 h), and the proteins indicated were detected by immunolocalization. Cytoplasmic condensates induced by PFT-µ accumulated the SG markers G3BP1 and HuR. (**B**–**D**) HeLa cells were treated with vehicle or PFT-µ (50 µM, 2 h). (**B**) The treatment with vehicle or PFT-µ was conducted in the absence or presence of 10 µg/mL cycloheximide (CHX). G3BP1 provided the SG marker. (**C**) The translation initiation factor eIF4G and G3BP1 were visualized by immunofluorescence in cells incubated with vehicle or PFT-µ. (**D**) PolyA^+^-containing RNA and G3BP1 were detected with a combination of in situ hybridization with oligo-dT50 and immunostaining. All scale bars are 20 µm.

Our initial immunofluorescence studies revealed that HeLa cells incubated with PFT-µ produced cytoplasmic foci. The foci accumulated the SG markers G3BP1 and HuR, demonstrating that PFT-µ stimulated the formation of SG-like compartments (Figure 1A). Consistent with established SG properties [75], the granules did not accumulate hsp70. To substantiate further that PFT-µ stimulates the generation of *bona fide* SGs, we examined additional characteristics of genuine SGs (Figure 1B–D). As such, cycloheximide abolished the PFT-µ-dependent production of cytoplasmic granules (Figure 1B). Further consistent with authentic SGs [76], PFT-µ-induced cytoplasmic granules that contain the translation initiation factor eIF4G (Figure 1C) and polyA^+^-containing RNA (Figure 1D). Taken together, these results show that PFT-µ triggered the formation of *bona fide* SGs.

### 3.2. SG Formation with Pifithrin-µ Requires the Phosphorylation of eIF2α

**Figure 2:**
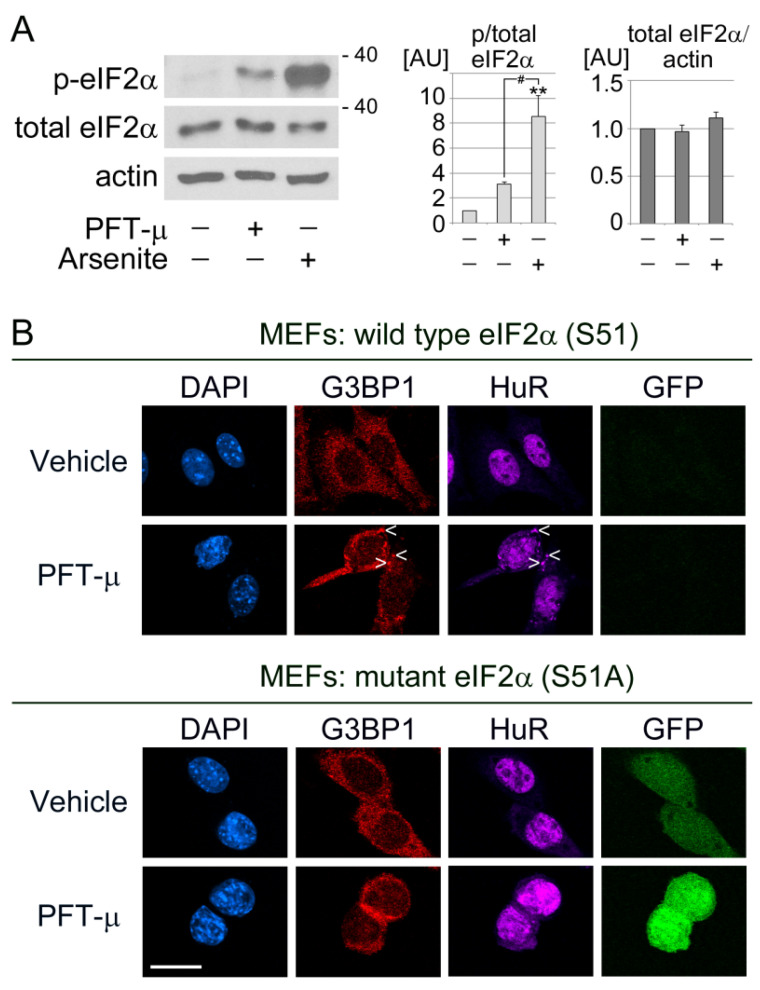
The formation of PFT-µ SGs relies on eIF2α phosphorylation. (**A**) HeLa cells were incubated with vehicle, PFT-µ, or arsenite. Crude extracts were evaluated for the phosphorylation of eIF2α on S51 (p-eIF2α) and total eIF2α. Actin provided a reference for loading. The molecular mass of marker proteins is indicated in kD at the right margin. The relative phosphorylation of eIF2α (p/total eIF2α) and the abundance of total eIF2α were quantified for at least three independent experiments. Results normalized to vehicle controls are depicted as average + standard error of the mean (SEM). One-way ANOVA combined with Bonferroni post hoc analysis identified significant differences between groups. Comparison to vehicle: **, *p* < 0.01. Comparison between PFT-µ and arsenite, # *p* < 0.05. (**B**) Wildtype and mutant mouse embryonic fibroblasts (MEFs) were incubated with PFT-µ. The formation of SGs was assessed with the SG marker proteins G3BP1 and HuR. Cells that produce mutant eIF2α (S51A) also synthesize GFP (Materials and Methods). All images were acquired with identical settings; scale bar is 20 µm. Several SGs are marked with arrowheads. Note that MEFs with mutant eIF2α do not generate SGs when treated with PFT-µ.

Many stressors stimulate the phosphorylation of translation initiation factor eIF2α on serine 51 (S51), which serves as an upstream signaling event to trigger SG formation [77]. However, SGs can also form independently of eIF2α phosphorylation [78,79]. To begin to define the molecular mechanisms through which PFT-µ promotes SG formation, we examined how the compound affects the phosphorylation of eIF2α on S51. Arsenite treatment (0.5 mM, 30 min) was used as positive control, as it elevates eIF2α phosphorylation. Figure 2A reveals that PFT-µ increased the S51 phosphorylation of eIF2α. By contrast, the abundance of total eIF2α remained largely unaffected.

We next determined whether S51 phosphorylation was necessary to generate PFT-µ SGs. This was accomplished with mouse embryonic fibroblasts (MEFs) engineered to synthesize the non-phosphorylatable S51A mutant of eIF2α. In Figure 2B, cells producing the S51A eIF2α mutant are identified with the fluorescent marker GFP [62]. (Note that the cells produce S51A eIF2α and GFP as separate proteins, not as a fusion protein). These mutant MEFs failed to form SGs upon PFT-µ treatment (Figure 2B). By contrast, PFT-µ clearly generated SGs in the cytoplasm of wild type MEFs. In summary, PFT-µ stimulated eIF2α phosphorylation on S51; this was a mandatory step to assemble PFT-µ SGs. Our results also demonstrate that PFT-µ caused SG assembly in cell lines derived from cancer cells as well as non-malignant fibroblasts.

### 3.3. The Properties of Pifithrin-µ-Induced SGs Change in a Time-Dependent Fashion

**Figure 3:**
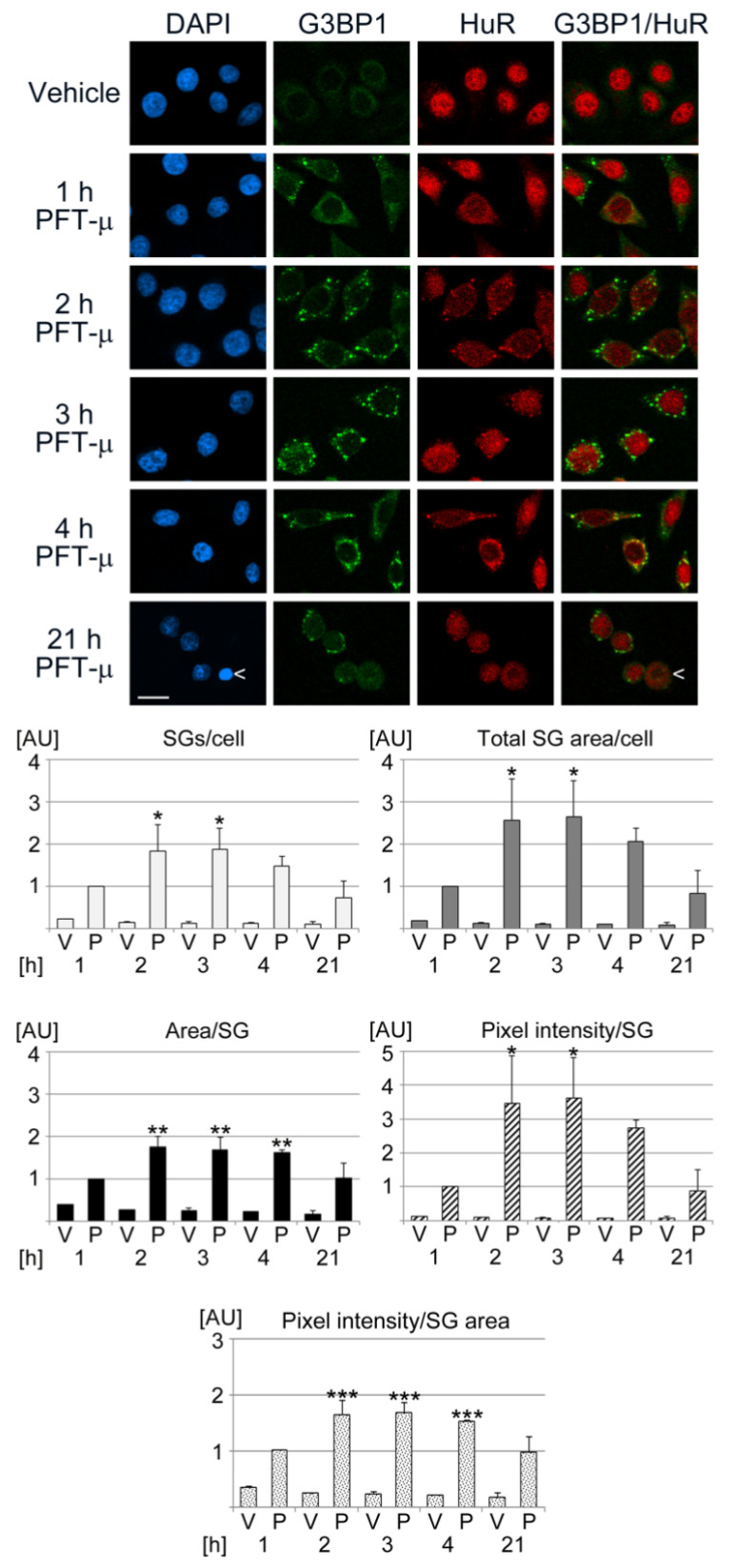
Kinetics of SG formation in response to PFT-µ treatment. HeLa cells were incubated with the vehicle DMSO (V) or PFT-µ (P) for the times indicated [h]. Scale bar is 20 µm. The arrowhead points to a pyknotic nucleus, which indicates cell death. For the measurements of SG properties, all images were acquired with identical settings. SG parameters were quantified for two independent experiments, each set with at least 112 cells per condition for each experiment. Results were normalized to the 1 h PFT-µ datapoint. Bar graphs depict data as average + SEM. Statistical evaluation was performed with one-way ANOVA and Bonferroni post hoc analysis. The 1 h vehicle control was used as reference for pairwise comparisons; *, *p* < 0.05; **, *p* < 0.01; ***, *p* < 0.001. AU, arbitrary units.

To characterize the SGs generated with PFT-µ, we determined the kinetics of granule formation (Figure 3). To this end, HeLa cells were assessed at different time points of PFT-µ treatment. Granule production was monitored with two SG markers, G3BP1 and HuR. During PFT-µ incubation, SGs were generated as early as 1 h. The granules increased further in size (area/SG) when the incubation time was extended to 4 h. A similar time course was observed for the number of SGs/cell, total SG area/cell, pixel intensity/SG, and pixel intensity/SG area. However, prolonged exposure to PFT-µ (21 h) reduced the values for all SG parameters. At the same time, cells displayed alterations in morphology, and pyknotic nuclei became more abundant (Figure 3, arrowhead). The nuclear accumulation of HuR was not static during the incubation period; these data are consistent with the nucleocytoplasmic shuttling and subcellular relocation of HuR reported by others [80]. Together, the experiments show that all monitored SG properties were dynamic and determined by the duration of PFT-µ exposure. The changes observed after 21 h exposure to PFT-µ were consistent with elevated cell death.

**Figure 4:**
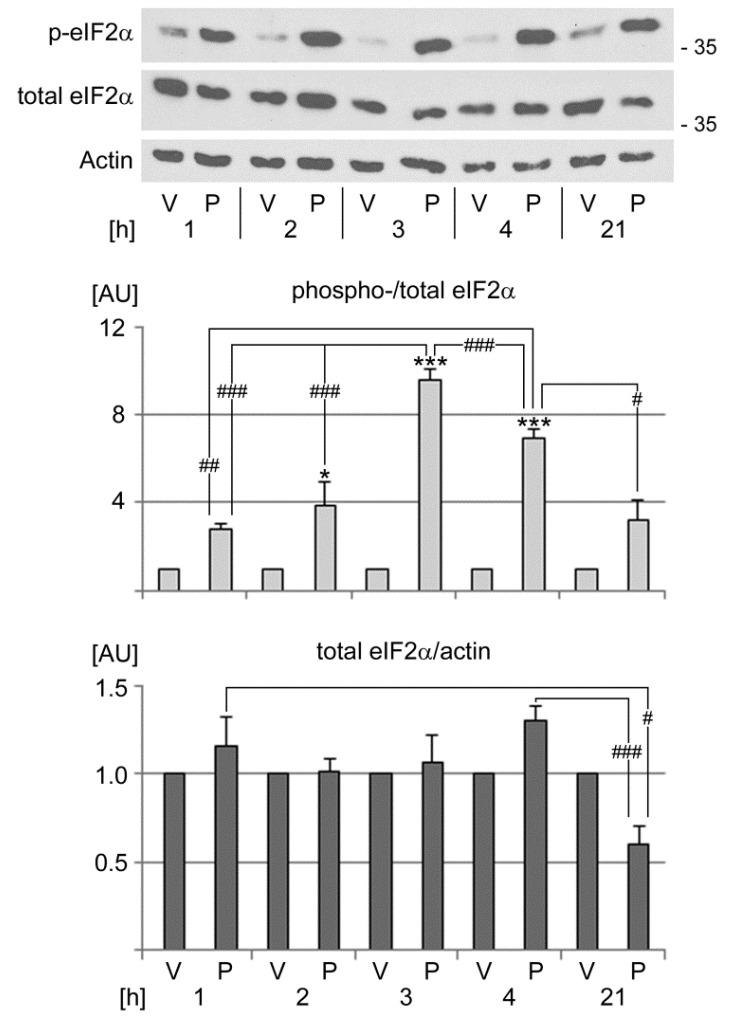
PFT-µ elevates the phosphorylation of eIF2α in a time-dependent fashion. HeLa cells were incubated with vehicle (V) or PFT-µ (P) for the hours [h] indicated. Crude extracts were assessed for eIF2α phosphorylation on S51 (p-eIF2α) and total eIF2α. Actin provided a loading reference. The molecular mass of marker proteins is shown in kD at the right margin. ECL signals were measured and normalized to the vehicle control for each time point. Two independent experiments were evaluated for p-eIF2α, and at least three independent experiments were performed for total eIF2α. Results are shown as average + SEM. Statistical evaluation was performed with one-way ANOVA, followed by Bonferroni post hoc analysis. Significant differences were identified relative to the vehicle control (*, *p* < 0.05; ***, *p* < 0.001). Pairwise comparisons showed significant differences between PFT-µ-treated samples (#, *p* < 0.05; ##, *p* < 0.01; ###, *p* < 0.001). AU, arbitrary units.

To define the signaling events that drive PFT-µ-mediated SG assembly, we monitored the S51 phosphorylation status of eIF2α at different time points. PFT-µ stimulated eIF2α phosphorylation in a time-dependent fashion (Figure 4). Moreover, the rise in eIF2α phosphorylation at 2, 3, and 4 h coincided with maximum SG formation (Figure 3). PFT-µ slightly increased the abundance of total eIF2α at 1 and 4 h of treatment, but diminished total eIF2α levels after 21 h.

### 3.4. Pifithrin-µ Diminishes the Abundance of SG Nucleators G3BP1 and TIA-1/TIAR

**Figure 5:**
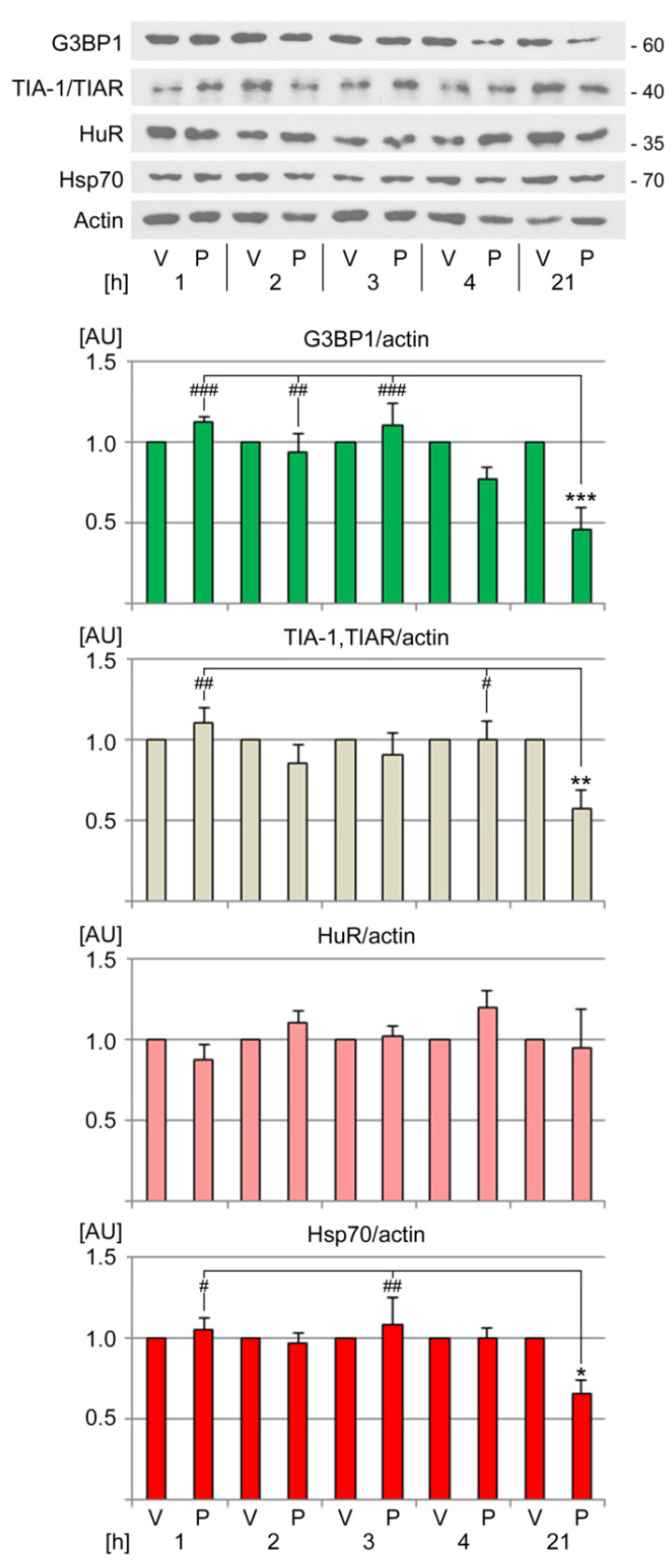
Impact of PFT-µ treatment on the abundance of SG nucleators, HuR, and hsp70. The levels of different SG components and hsp70 were determined by Western blotting as described for Figure 4. The molecular mass of marker proteins is depicted in kD at the right margin. Three to four independent experiments were conducted for each protein analyzed. Graphs show averages + SEM. Statistical evaluation was conducted with one-way ANOVA and Bonferroni post hoc analysis. Significant differences are indicated relative to the vehicle control (*, *p* < 0.05; **, *p* < 0.01, ***, *p* < 0.001). Pairwise comparisons identified significant differences relative to the 21 h PFT-µ treatment (#, *p* < 0.05; ##, *p* < 0.01; ###, *p* < 0.001). V, vehicle; P, PFT-µ; AU, arbitrary units.

Experiments in Figure 4 linked the dynamics of PFT-µ-induced eIF2α phosphorylation to the generation of SGs. To identify additional mechanisms contributing to the time-dependent changes in SG characteristics, we measured the abundance of the SG nucleators G3BP1 and TIA-1/TIAR. The SG marker HuR and hsp70, the chaperone targeted by PFT-µ, were also examined (Figure 5). The rationale for these experiments was our previous observation that the loss of SG nucleators diminishes SG size [81]. After 21 h PFT-µ incubation, the levels of G3BP1, TIA-1/TIAR, and hsp70 were significantly reduced. Notably, no marked changes were observed for HuR. Together, our data are consistent with the interpretation that multiple factors contribute to the decrease in SG number and size at the 21 h time point. These factors include the diminished phosphorylation of eIF2α (Figure 4) and the reduced abundance of SG nucleators (Figure 5). Notably, the loss of SGs upon prolonged PFT-µ treatment may compromise cell viability [5]. Indeed, cell survival declined after an extended period of PFT-µ incubation (21 h), as discussed in the next section.

### 3.5. Prolonged Pifithrin-µ Treatment Reduces Cell Viability

The formation of SGs is linked to the survival of stress ([1] and references therein). Accordingly, the time-dependent decline in PFT-µ SGs (Figure 3) may be associated with a loss of cell viability. To address this question, we monitored cell metabolic activities with CellTiter-Blue^®^, a resazurin reduction assay. Resazurin is only cleaved by viable, metabolically active cells to resorufin, a fluorescent compound [82].

**Figure 6:**
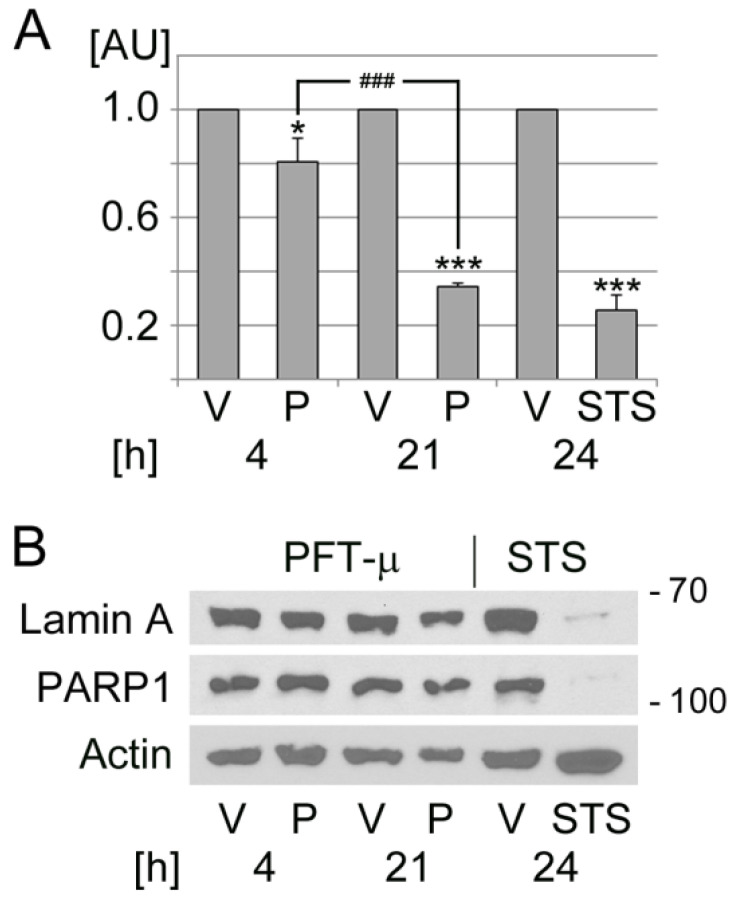
PFT-µ induces cell death in a time-dependent fashion. HeLa cells were incubated with vehicle (V), PFT-µ (P), or staurosporine (STS) for the hours [h] indicated. (**A**) PFT-µ reduced the metabolic activity of HeLa cells. Results for each condition were normalized to the vehicle control. Data are depicted as average + SEM for three independent experiments. One-way ANOVA combined with Bonferroni post hoc analysis was used for statistical evaluation. The vehicle control served as reference. *, *p* < 0.05; ***, *p* < 0.01. Pairwise comparison demonstrated significant differences between 4 h and 21 h PFT-µ treatments; ###, *p* < 0.001. AU, arbitrary units. (**B**) Crude extracts were prepared for HeLa cells that were treated as described for part A. Western blotting evaluated the cleavage of lamin A and PARP1. Actin served as loading reference. The molecular mass of marker proteins is shown in kD at the right margin.

As shown in Figure 6A, PFT-µ diminished HeLa cell viability in a time-dependent fashion. A 4 h incubation period reduced cell viability to ~80%, which was further decreased to ~34% after 21 h. Staurosporine was included as a positive control, as it induces morphological changes and apoptosis in mammalian cells [63,83]. After 24 h, staurosporine reduced the metabolic activity of HeLa cells to ~25% of the vehicle controls (Figure 6A).

PFT-µ can cause cell death through different pathways [33,34,70]. To monitor the possible contribution of apoptosis, the cleavage of lamin A and PARP1 was assessed (Figure 6B). There was no pronounced effect of PFT-µ on lamin A and PARP1 abundance. By contrast, staurosporine resulted in a marked reduction of both proteins, which indicates staurosporine-induced apoptotic cell death.

In summary, our experiments revealed that (i) PFT-µ caused time-dependent changes related to the presence and properties of SGs. While short-term incubation triggered a stress response associated with SG formation, long-term treatment diminished the number of SGs/cell and altered all SG parameters quantified by us. (ii) Long-term incubation with PFT-µ led to a significant loss of cell viability. (iii) The changes in metabolic activity triggered by PFT-µ did not correlate with a marked cleavage of lamin A or PARP1. These results support the interpretation that PFT-µ induced death predominantly through non-apoptotic routes. Our data are in line with unrelated studies that report non-apoptotic cell death upon treatment with PFT-µ [33,34]. While PFT-µ is linked to necroptotic cell death [33], the current study did not assess necroptotic mediators in treated cells. To uncover the type(s) of cell death instigated by long-term PFT-µ exposure, future experiments will have to evaluate biomarkers of necroptosis and other forms of regulated cell death [84,85].

### 3.6. Pifithrin-µ Treatment Significantly Reduces AMPK Activation

**Figure 7:**
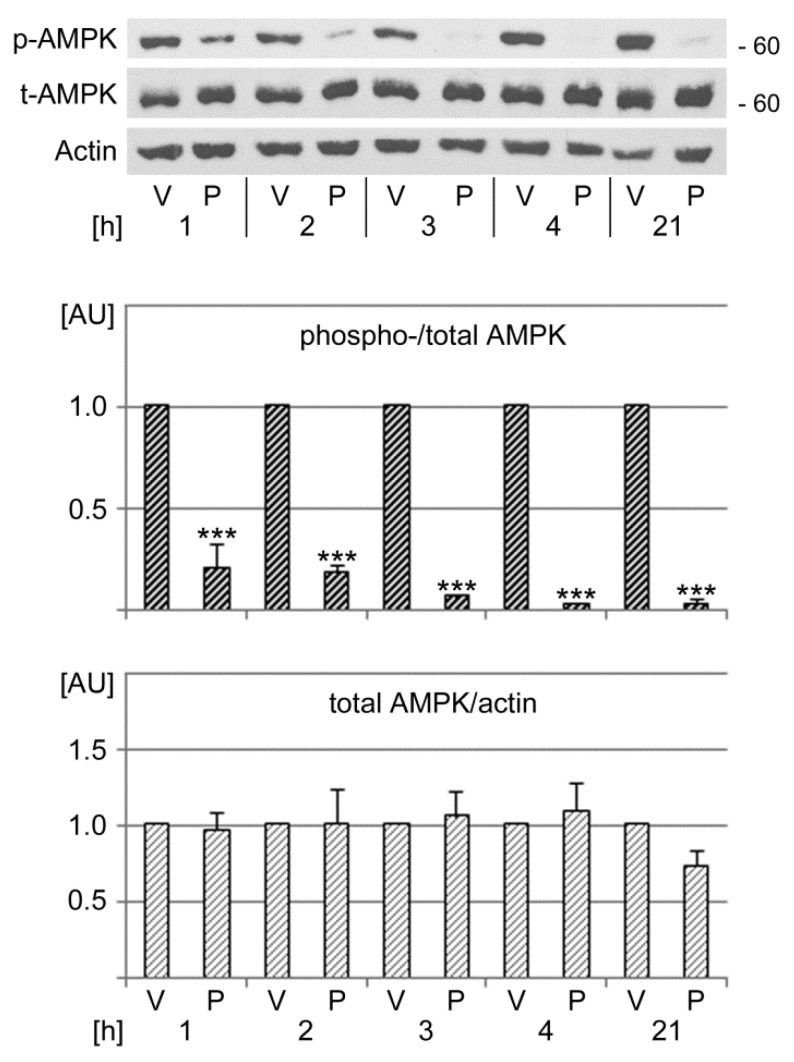
PFT-µ diminishes the phosphorylation of AMPK on T172. Cells were incubated with vehicle (V) or PFT-µ for the hours [h] depicted. Crude extracts were evaluated for the phosphorylation of AMPK on T172 (p-AMPK). The same samples were also probed with antibodies against total AMPK (t-AMPK) and actin. The molecular mass of marker proteins in kD is indicated at the right margin. ECL signals were quantified for three independent experiments. Data were normalized to vehicle controls for each time point. Bars show average + SEM for each datapoint. Statistical evaluation was performed with one-way ANOVA combined with Bonferroni post hoc analysis. Changes were assessed relative to the vehicle control; ***, *p* < 0.001. AU, arbitrary units.

AMPK functions as a stress sensor, and our earlier work showed that the kinase directly modulates SG biogenesis [5,9]. These previous insights prompted us to examine the effects of PFT-µ on AMPK signaling. To this end, we assessed the phosphorylation of AMPK on T172 of the catalytic α-subunit, which represents an important step to fully activate the kinase [86]. Western blotting demonstrated that PFT-µ significantly reduced AMPK activation throughout the incubation period (Figure 7). By contrast, the abundance of total AMPK did not significantly change over the time course of the experiment.

Collectively, our results show that PFT-µ downregulates signaling through AMPK. The profound reduction in AMPK phosphorylation on T172 during PFT-µ treatment is accompanied by the loss of cell viability. These data substantiate and extend our previous and unrelated work. Specifically, we have demonstrated earlier that AMPK activation enhances the survival of oxidative stress. Furthermore, oxidative and other stressors can diminish AMPK phosphorylation on T172 [5,9,65]. Figure 6 and Figure 7 corroborate these links, as PFT-µ caused a simultaneous loss of cell viability and AMPK activation.

We have shown previously that AMPK activity regulates SG formation [5,9,87]. Our work demonstrated that AMPK activation modulates eIF2α phosphorylation; the kinase also controls SG properties through its effect on core SG proteins and microtubules. Based on these observations, AMPK is expected to contribute to multiple cellular responses to PFT-µ. Ultimately, these responses determine cell fate decisions and fine-tune SG properties.

### 3.7. Pifithrin-µ Treatment Activates the Pro-Survival Kinase Akt

The crosstalk between AMPK and Akt signaling is well-documented [56,57,58,59], and the pivotal role of Akt in cancer cell survival is firmly established [53,54,55]. The kinase activity of Akt is regulated by two major posttranslational modifications. T308 phosphorylation is mandatory to activate the kinase; the kinase activity is further enhanced by S473 phosphorylation [55]. Notably, S473 phosphorylation limits oxidant-induced apoptosis [88] and promotes adhesion-dependent survival [89].

**Figure 8:**
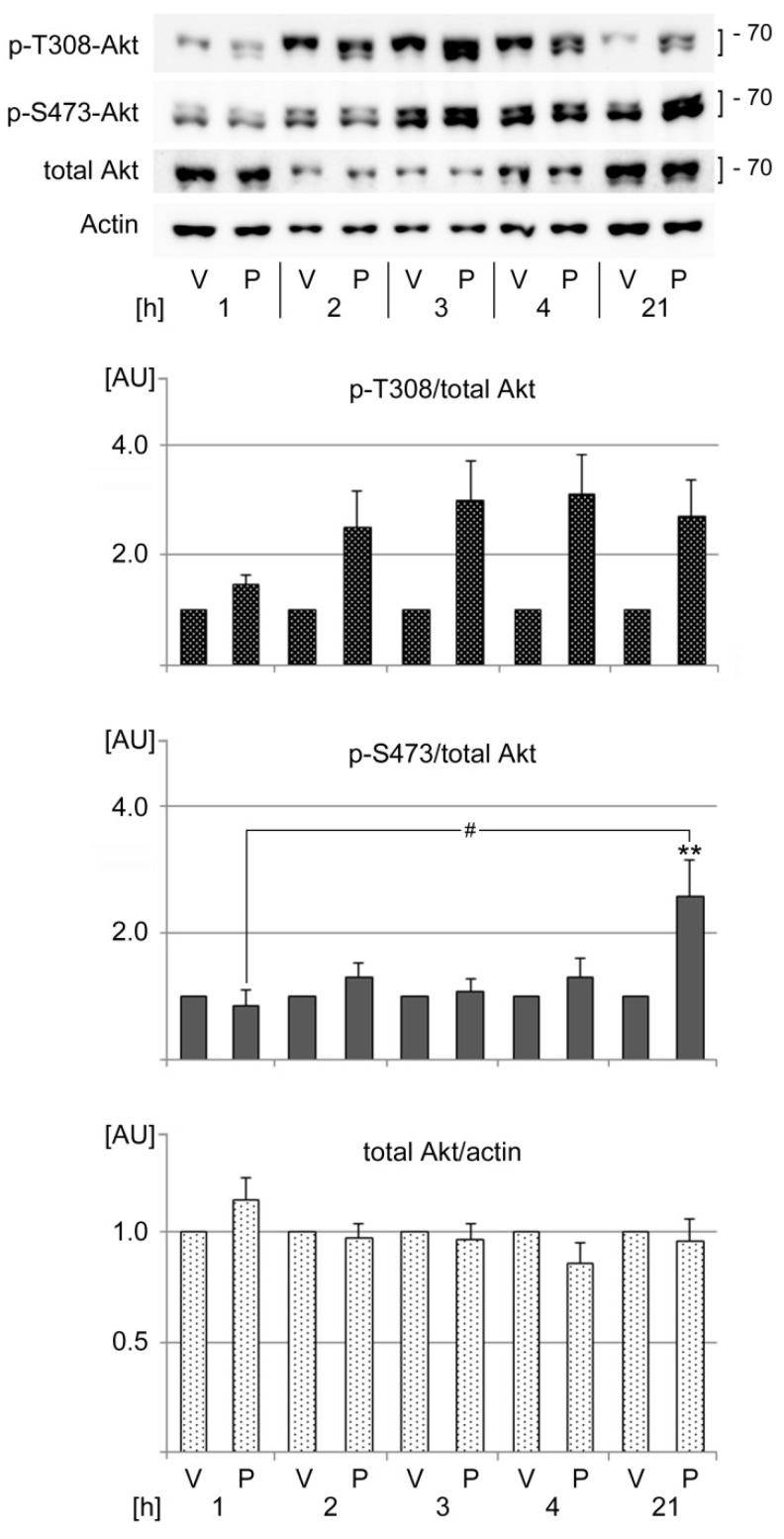
PFT-µ modulates Akt activation. HeLa cells were incubated with vehicle (V) or PFT-µ (P) for the hours [h] specified. Crude extracts were evaluated for Akt phosphorylation on T308 (p-T308) or S473 (p-S473) and for total Akt. The bands quantified for p-T308, p-S473, and total Akt are marked with square brackets. Actin was used as loading reference. The molecular mass of marker proteins is indicated at the right margin in kD. ECL signals were quantified and normalized to the vehicle control for each time point. Results for four to seven independent experiments are depicted as average + SEM. Statistical evaluation was performed with one-way ANOVA followed by Bonferroni post hoc analysis. Significant differences were identified relative to the vehicle control (**, *p* < 0.01). Pairwise comparisons between PFT-µ-treated samples showed a significant difference for p-S473 (#, *p* < 0.05). AU, arbitrary units.

Directly relevant to our study, signaling through the PI3 kinase pathway stimulates SG assembly and requires Akt phosphorylation on T308 [12]. This motivated us to examine the possible effects of PFT-µ on Akt phosphorylation and abundance. As illustrated in Figure 8, PFT-µ stimulated T308 phosphorylation, especially during prolonged treatment (>1 h). At the same time, SG numbers and sizes reached maximal values (Figure 3). Unlike T308, the phosphorylation of S473 increased significantly after 21 h incubation with PFT-µ. These data suggest that PFT-µ has different outcomes for the steady-state phosphorylation of T308 and S473.

The pro-survival signaling mediated by Akt is important for cell viability. The upregulation of S473 phosphorylation after 21 h of PFT-µ treatment may limit cell apoptosis. Whether the enhanced S473 modification during chronic PFT-µ stress restricts cell death will have to be investigated in the future.

## 4. Conclusions

**Figure 9:**
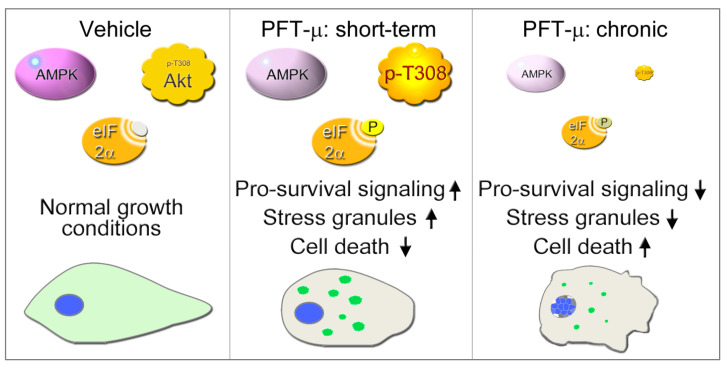
Simplified model of the cellular responses triggered by PFT-µ. Stress granules are depicted as green spherical compartments in the cytoplasm. Short-term refers to the incubation period up to 4 h; chronic represents a 21 h treatment. ↑, upregulation; ↓, downregulation. See text for details.

This study evaluated the impact of PFT-µ on cell physiology. To our knowledge, this is the first in-depth investigation that links PFT-µ to the formation of *bona fide* SGs and the signaling events that are relevant to granule assembly and cell viability (summarized in Figure 9). Here, we demonstrated that the SG formation triggered by PFT-µ was a dynamic process and required eIF2α phosphorylation. Time-dependent alterations in eIF2α phosphorylation were accompanied by changes in AMPK and Akt signaling; all of these processes control stress responses and cell survival. We uncovered significant differences between the initial responses to PFT-µ that lasted up to 4 h and the later effects that were uncovered at 21 h. While cells activated pro-survival pathways at the earlier time points and cell death was limited, prolonged exposure to PFT-µ profoundly altered SG properties and signaling, concomitant with an extensive loss of cell viability.

Together, our results support the model that different signaling pathways converge to control SG formation and cell survival when cells are treated with PFT-µ (Figure 9). We identified the following key events that are directly relevant to these processes: (i) The presence of SGs was most prominent between 2 and 4 h of PFT-µ incubation. (ii) PFT-µ elevated the phosphorylation of eIF2α, which peaked concomitantly with SG formation. (iii) The decline of SG-nucleators G3BP1 and TIA-1/TIAR at the 21 h time point coincided with reduced SG numbers and substantial cell death. (iv) PFT-µ diminished AMPK activation; this decline continued throughout the incubation period. (v) Akt phosphorylation on T308 followed similar kinetics as SG production and eIF2α phosphorylation on S51. (vi) Prolonged exposure to PFT-µ caused cell death. The loss of cell viability was at least in part mediated by non-apoptotic pathways.

The current study broadens the information on signaling events that regulate granulostasis. As such, both AMPK and Akt activities impinge on SG biogenesis. As described by us for PFT-µ, oxidative stress also inhibits the phosphorylation of AMPK on T172 [9]. Collectively, these results emphasize the interplay between AMPK and SG formation. On the other hand, Akt negatively regulates the expression of the *G3BP1* gene [90], which could limit SG formation at later time points. Consistent with this idea, PFT-µ reduced SG numbers and size after 21 h incubation when compared with earlier time points. Furthermore, PI3 kinase, which stimulates Akt phosphorylation on T308, may function as pro-SG kinase under oxidative stress conditions [12]. This observation is in line with our results, as Akt activation accompanied the production of PFT-µ SGs.

PFT-µ targets molecular chaperones of the hsp70 family, which are key components of the cellular proteostasis network and stress-related signaling. In the context of SGs, hsp70s control granule assembly, dynamics, and their removal during stress recovery [20]. Hence, the inhibition of hsp70 by PFT-µ may promote the interactions among granule-nucleation factors and, at the same time, limit the dissolution of SGs. Moreover, hsp70 associates with protein kinases and phosphatases that are implicated in granulostasis [91]. This includes subunits of AMPK and the protein phosphatase PP1, which dephosphorylates phospho-S51 of eIF2α [92]. Therefore, it is likely that hsp70 inhibition affects multiple aspects of granulostasis, either by direct binding of granule components or through regulating signaling events that control granule properties.

At present, it is not known how PFT-µ increases the phosphorylation of eIF2α. Possible factors contributing to this event include the eIF2α kinases PERK (PKR-like endoplasmic reticulum kinase), PKR (protein kinase double-stranded RNA-dependent), GCN2 (general control non-derepressible-2), and HRI (heme-regulated inhibitor). As well, PFT-µ may impinge on the phosphatase complexes that antagonize the action of eIF2α kinases [93]. These are interesting questions that need to be explored in the future.

In summary, our study provides novel insights into the modulation of stress-responsive pathways by PFT-µ (Figure 9). Our findings are important information for the development of therapeutic applications that explore molecular chaperones or other factors of the integrated stress response. Such regimens are clinically relevant to the treatment of various forms of cancer and other diseases [1,3,93,94].

## Figures and Tables

**Table 1:** Source of primary antibodies and the dilutions for Western blotting or immunolocalization. NA, not applicable.

Primary Antibodies				
Protein	Supplier	Catalog Number	Dilution for Western Blotting	Dilution for Immunolocalization
G3BP1 (mouse)	BD Biosciences, Franklin Lakes, NJ, USA	clone 23/G3BP1	1:1000	1:2000
G3BP1 (mouse)	Santa Cruz Biotechnology, Dallas, TX, USA	sc-365338	1:1000	1:500
G3BP1 (rabbit)	Bethyl Laboratories, Montgomery, TX, USA	A302-033A	NA	1:1000
HuR	Santa Cruz Biotechnology	sc-5261	1:2000	1:1000
p-eIF2α (S51)	Cell Signaling Technology, Withby, ON, Canada	#3597	1:1000	NA
Total eIF2α	Santa Cruz Biotechnology	sc-30882	1:1000	NA
TIA-1/TIAR	Santa Cruz Biotechnology	sc-28237	1:1000	NA
Hsp70 (hsp72)	Enzo Life Sciences, Toronto, ON, Canada	SPA-812	1:1000	NA
eIF4G	Cell Signaling	#2469	NA	1:250
Lamin A	Santa Cruz Biotechnology	sc-20680	1:1000	NA
PARP1	Santa Cruz Biotechnology	sc-25780	1:1000	NA
p-AMPK-α1/2	Cell Signaling	#2535	1:2000	NA
AMPK-α1/2	Cell Signaling	#2532	1:2000	NA
p-T308-Akt	Cell Signaling	#4056	1:2000	NA
p-S473-Akt	Santa Cruz Biotechnology	sc-7985	1:4000	NA
Akt	Cell Signaling	#9272	1:1500	NA
Actin	Chemicon, Temecula, CA, USA	mab1501	1:100,000	NA
**Secondary Antibodies**				
**Tag**		**Supplier**	**Dilution for** **Western blotting**	**Dilution for** **Immunolocalization**
Horseradish peroxidase (HRP)		Jackson ImmunoResearch, West Grove, PA, USA	1:2000	NA
Alexa Fluor^®^ 488, Cy™3, Alexa Fluor^®^ 647		Jackson ImmunoResearch	NA	1:200–1:500

## Data Availability

Analytic methods and materials will be made available upon publication of this article. The original data will be available from the corresponding author upon reasonable request.

## Supplementary Materials

The following supporting information can be downloaded at: https://www.mdpi.com/article/10.3390/cells13110885/s1, raw data for Western blotting.

## Abbreviations

AMPK: AMP-activated protein kinase; ECL: enhanced chemiluminescence; Hsp70: heat shock protein 70; MEF: mouse embryonic fibroblast; PARP1: poly[ADP-ribose] polymerase 1; PFT-µ: pifithrin-µ; SG: stress granule.
