# Supplementary material for: Pifithrin-µ Induces Stress Granule Formation, Regulates Cell Survival, and Rewires Cellular Signaling
Source: Cells. 2024 May 21;13(11):885. doi: 10.3390/cells13110885 (PMC11172192; doi:10.3390/cells13110885)

Supplementary Information

Pifithrin-μ induces stress granule formation, regulates cell survival and rewires cellular signaling

Hicham Mahboubi[1], Henry Yu[1], Michael Malca[1], David McCusty[1] and Ursula Stochaj[1, 2, *]

[1]Department of Physiology, McGill University, Montreal, Quebec, Canada

[2]Quantitative Life Sciences Program, McGill University

Raw data for Western blots.

The original films for enhanced chemiluminescence are provided below for all figures. The related figure in the main text is shown on top of the raw data. If films include results for different experiments, a black line on top of the filter indicates lanes that are pertinent to the main manuscript. For Fig. 5 and TIA-1/TIAR, the position of the relevant band is marked with an arrowhead. For Fig. 8 and total Akt, the positions of total Akt and actin are indicated at the right margin.

Figure 2

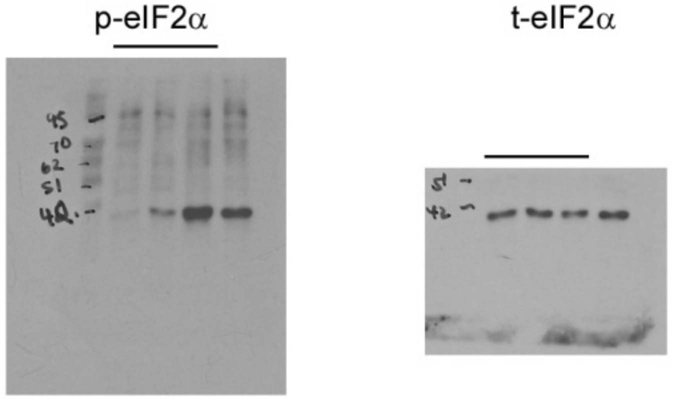

Figure 4

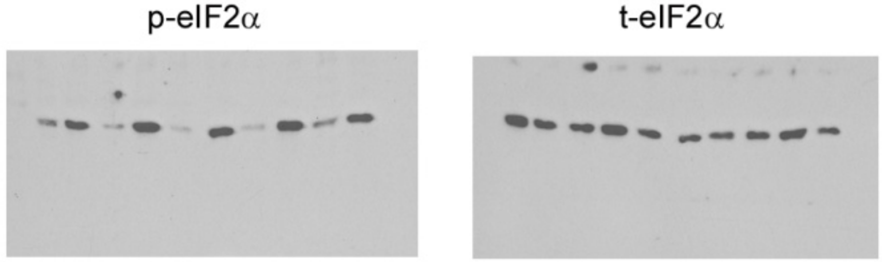

Figure 5

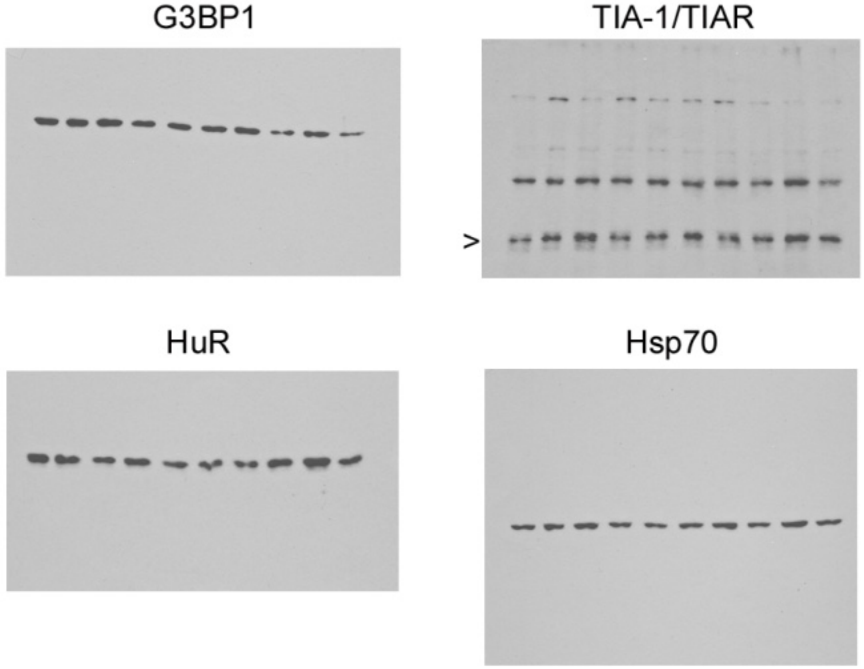

Figure 6

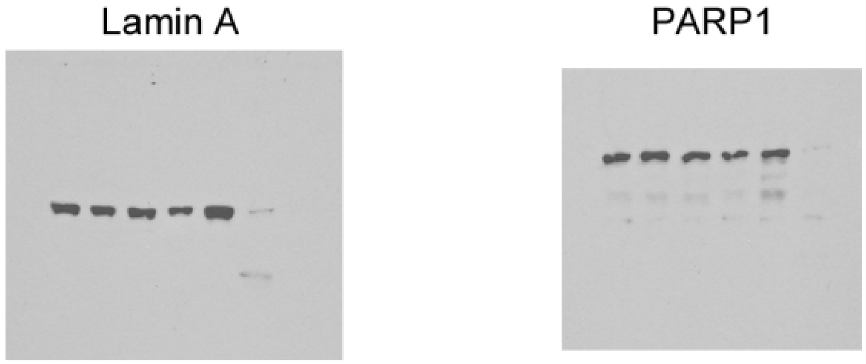

Figure 7

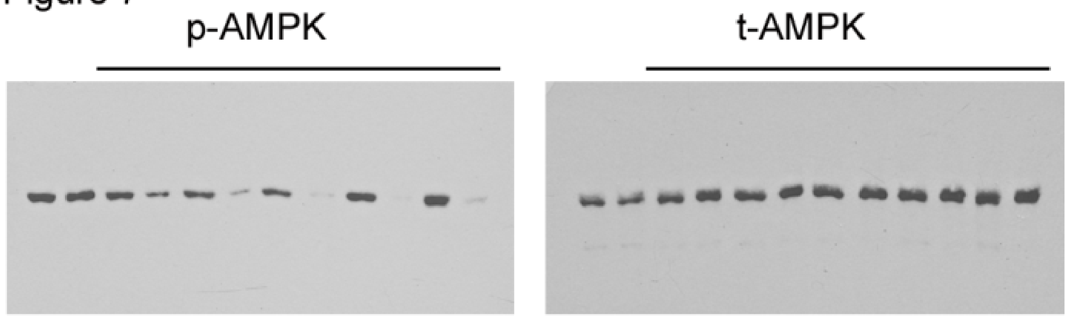

Figure 8

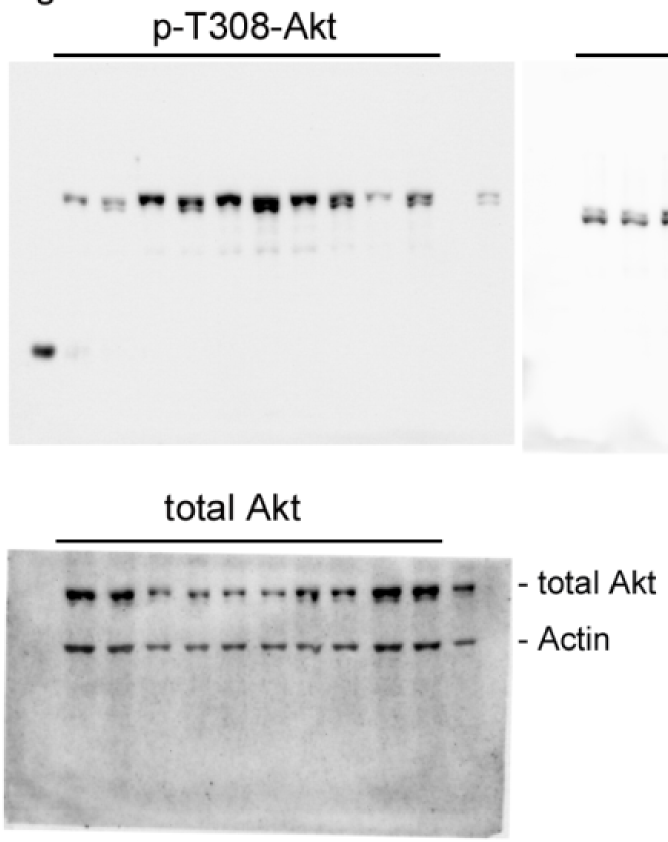

Supplement: Supplementary file 1 [file cells-13-00885-s001.zip › cells-2810205-supplementary.pdf]
